# Role of the Nrf2-ARE Pathway in Liver Diseases

**DOI:** 10.1155/2013/763257

**Published:** 2013-05-09

**Authors:** Sang Mi Shin, Ji Hye Yang, Sung Hwan Ki

**Affiliations:** College of Pharmacy, Chosun University, Seosuk-dong, Dong-gu, Gwangju 501-759, Republic of Korea

## Abstract

The liver is a central organ that performs a wide range of functions such as detoxification and metabolic homeostasis. Since it is a metabolically active organ, liver is particularly susceptible to oxidative stress. It is well documented that liver diseases including hepatitis, fibrosis, cirrhosis, and hepatocellular carcinoma are highly associated with antioxidant capacity. NF-E2-related factor-2 (Nrf2) is an essential transcription factor that regulates an array of detoxifying and antioxidant defense genes expression in the liver. It is activated in response to electrophiles and induces its target genes by binding to the antioxidant response element (ARE). Therefore, the roles of the Nrf2-ARE pathway in liver diseases have been extensively investigated. Studies from several animal models suggest that the Nrf2-ARE pathway collectively exhibits diverse biological functions against viral hepatitis, alcoholic and nonalcoholic liver disease, fibrosis, and cancer via target gene expression. In this review, we will discuss the role of the Nrf2-ARE pathway in liver pathophysiology and the potential application of Nrf2 as a therapeutic target to prevent and treat liver diseases.

## 1. Introduction 

The liver is a multifunctional organ that is responsible for detoxification and metabolic homeostasis. It has two blood supply sources: the hepatic artery delivers oxygenated blood from the general circulation and the portal vein supplies deoxygenated but nutrient-rich blood from the intestinal region [1]. Many cell types compose the liver. The parenchymal cells, which are the most abundant in the liver, are hepatocytes (80% by volume) [2]. The nonparenchymal cells such as endothelial cells, Kupffer cells, smooth muscle cells, hepatic stellate cells, and oval cells are other important cell components in the liver [2]. All of these cells can modulate the progression of liver diseases and activate multiple signaling pathways. 

 The liver is the first organ exposed to orally administered xenobiotics after absorption from the intestine, and it is a major site of biotransformation and metabolism. Since the liver is a metabolically active organ, it is particularly susceptible to reactive oxygen species (ROS). ROS are produced in liver cells as byproducts of normal metabolism and detoxification. Therefore, a wide range of antioxidant systems have developed in the liver, so that when produced, ROS are rapidly destroyed [3]. However, sustained and excessive ROS cause cellular damage and have been linked to a variety of liver diseases. Viral hepatitis and alcoholic or nonalcoholic steatohepatitis are the 3 major causes of chronic liver diseases, which are highly associated with oxidative stress, lead to liver fibrosis, cirrhosis, and end-stage hepatocellular carcinoma (HCC). Therefore, it is generally accepted that oxidative stress plays a key role in promoting the progression of these liver diseases [4].

 Elevated ROS and electrophiles induce a series of antioxidant genes through the activation of antioxidant response element (ARE) to protect cells against oxidative stress [5]. ARE-containing gene expression is primarily regulated by NF-E2-related factor-2 (Nrf2), a member of the cap'n'collar family of bZIP transcription factors [6]. Nrf2 is activated in response to oxidative stress and electrophiles in a variety of tissues and cells and plays a role as a multiorgan protector through target gene induction [7]. Keap1 is a negative regulator of Nrf2 and acts as an adaptor protein for functional E3 ubiquitin ligase complex with Cul3 and Rbx1 [8, 9]. In agreement with that, Nrf2 is constitutively accumulated in nuclei in Keap1-knockout mice [10]. 

**Figure 1:**
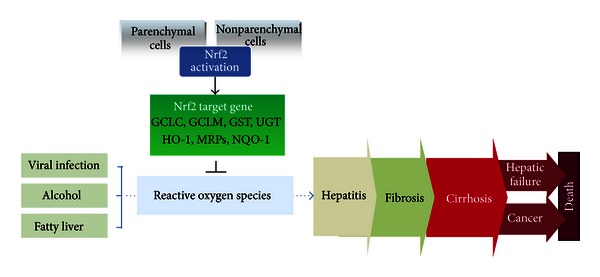
The protective role of Nrf2 activation in liver disease. This scheme shows that the activation of Nrf2 in hepatic parenchymal and nonparenchymal cells may alleviate various liver diseases through the inhibition of ROS production.

 Nrf2 activation is observed in nonparenchymal cells including hepatic stellate cells and Kupffer cells as well as in parenchymal hepatocytes [11, 12]. Moreover, many kinds of Nrf2 target genes are also expressed in the liver. Nrf2 plays complex and multicellular roles in hepatic inflammation, fibrosis, hepatocarcinogenesis, and regeneration via its target gene induction (Figure 1). Therefore, the protective roles of Nrf2 activation in the pathogenesis of liver diseases have been extensively investigated. Nrf2-knockout mice and Keap1-knockout mice have been made available and used extensively to investigate the functions of Nrf2 in many hepatic injury models. In this review, we highlight recent advances in Nrf2 signaling in liver pathophysiology and discuss the potential application of Nrf2 as a therapeutic target to prevent and to treat liver diseases.

## 2. Role of Nrf2 in Viral Hepatitis

 Viral hepatitis is a common infectious disease worldwide. There are at least six main hepatitis viruses, referred to as types A, B, C, D, E, and G, but types B and C viruses are more prevalent and might lead to liver cirrhosis and eventually cancer. An estimated 350 million people worldwide and 1.4 million people in the United States have chronic hepatitis B virus (HBV) infection [13]. An estimated 250 million people worldwide and 2.7 million people in the United States have chronic hepatitis C virus (HCV) infection [13]. Chronic viral hepatitis may lead to cirrhosis in about 20% of infected patients. Although the incidence of chronic HBV and HCV varies depending on several factors, it is well known that both infectious diseases are associated with oxidative stress [14]. However, only limited studies to date have examined the impact of Nrf2 on viral hepatitis, because hepatitis virus only infects humans and chimpanzees, having virtually no effect in other species, meaning conventional rat or mouse animal models are ineffective. Further studies are needed to identify the role of Nrf2 in viral hepatitis patient samples or an adequate animal model.

### 2.1. Nrf2 Signaling in HCV

**Figure 2:**
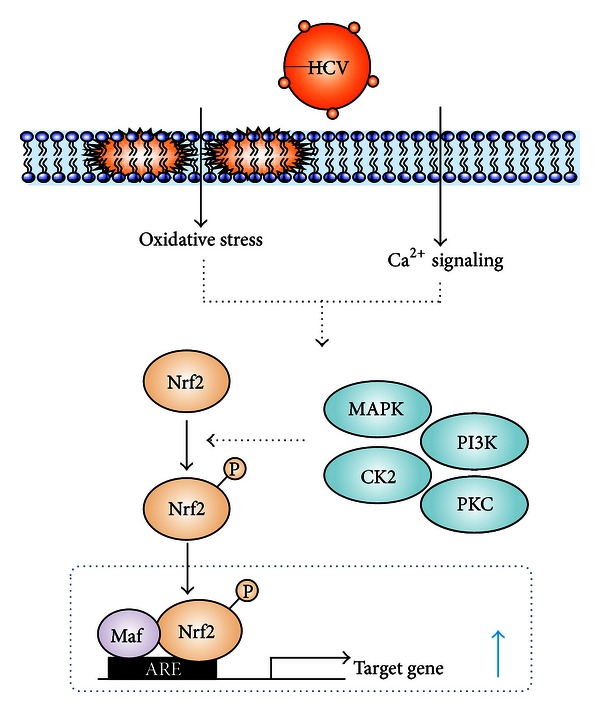
Signaling pathway in Nrf2 activation by HCV infection. Oxidative stress and Ca^2+^ mobilization induced by HCV infection activate various kinases, resulting in Nrf2 activation.

 The HCV genome is a single-stranded positive-sense RNA molecule of ~9,600 bases in length and encodes a large polyprotein, that is, cleaved into 11 structural (core, E1 and E2) and six nonstructural (NS) proteins (NS2-NS5B) [15]. HCV is associated with oxidative stress in human liver cells as indicated by oxidative stress markers, such as malondialdehyde, nitric oxide, and myeloperoxidase activity, which are much higher in chronic HCV patients than in healthy control subjects [16]. HCV gene expression is also reported to induce oxidative stress through Ca^2+^ signaling in the endoplasmic reticulum [17–19]. HCV infection in Huh-7 cells, a human hepatocarcinoma cell line, increases nuclear translocation of Nrf2 in a time-dependent manner and Nrf2-dependent gene induction, which contribute to cell survival against HCV infection [20]. Mitogen-activated kinases, casein kinase 2, phosphoinositide-3 kinase, and protein kinase C are involved in the phosphorylation and subsequent nuclear translocation of Nrf2 in HCV-infected cells [20, 21] (Figure 2). Consistent with this, HCV-induced Nrf2 activation is abrogated in the presence of antioxidants or Ca^2+^ chelators [20]. However, Carvajal-Yepes et al. recently reported that HCV impaired the induction of Nrf2-ARE-regulated genes by increasing the amount of small Maf proteins [22], which negatively regulate the expression of Nrf2-ARE-mediated genes [23]. Colocalization and direct interactions between small Maf and the nonstructural protein NS3, but not structural core proteins, are observed in HCV-replicating cells [22]. The controversial reports on the effect of Nrf2 activation in the antiviral defense might be due to the difference in the experimental design. Burdette et al. mainly focused on the basal level of Nrf2 translocation and target gene expression after HCV infection, whereas Carvajal-Yepes et al. evaluated the effect of HCV infection on Nrf2 overexpression- or tert-butylhydroquinone-induced Nrf2 activation. 

### 2.2. Nrf2 Signaling in HBV

HBV is a circular DNA virus of the Hepadnaviridae family, and the HBV genome encodes two regulatory proteins: PreS2 activator large surface protein (LHBs) and the Hepatitis B virus X protein (HBx). Both proteins regulate a series of different intracellular signaling cascades [24]. Schaedler et al. recently showed that in a human HBV genome introduced-stable cell line, HepAD38 or HepG2.2.15, the expression of diverse cytoprotective genes that are regulated by Nrf2-ARE pathway *in vitro* and *in vivo* was increased compared with that in HBV-negative HepG2 cells. The HBV-mediated gene induction is primarily initiated by the two regulatory proteins of HBV, HBx and LHBs, and is triggered by the kinases, c-Raf and MEK. The Nrf2 activation results in better protection of HBV-infected cells against oxidative damage as compared with that in control cells. Moreover, HBV infection in the cells increased the Nrf2-regulated proteasomal subunit PSMB5, and HBV-positive cells have higher constitutive proteasome activity and decreased immunoproteasome activity compared with control cells. However, Nrf2 activation does not affect HBV replication [25]. 

## 3. Role of Nrf2 in Drug- or Chemical-Induced Hepatitis

 The liver plays a major role in metabolizing xenobiotics such as alcohol, drugs, chemicals, and toxins. It breaks them down and eliminates them in separate steps, called phase I and phase II metabolism. The metabolism of xenobiotics could generate highly reactive intermediates which reduce molecular oxygen directly to produce ROS. Hundreds of drugs, chemicals, and toxins can cause reaction in the liver and lead to damage, that is, similar to that in acute viral hepatitis. 

### 3.1. Nrf2 Signaling in Acetaminophen- (APAP-) Induced Hepatotoxicity

**Figure 3:**
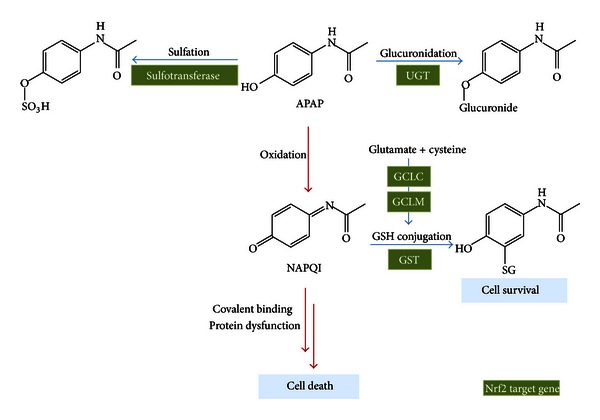
The role of Nrf2 target genes in APAP metabolism. Nrf2 transcriptionally regulates a variety of genes including sulfotransferase, UGT, GCLC, GCLM, and GST, which results in metabolic inactivation of APAP.

Drug-induced liver injury is a significant public health problem, accounting for over half of all cases of acute liver failure [26]. Worldwide, APAP is one of the most widely used nonprescription drugs for its analgesic and antipyretic activities. Even though it is a safe drug at a normal therapeutic dose, the possibility of hepatotoxicity remains. Indeed the major cause of drug-induced liver failure and death in the United States is APAP overdose poisonings as evidenced by over 100,000 cases each year [27, 28]. When used at therapeutic doses, APAP is primarily metabolized in the liver by glucuronidation (52–57% total urinary metabolites) catalyzed by UDP-glucuronosyltransferase (UGT) and sulfation (30–44%) by sulfotransferases and oxidation (<5%) [29, 30]. When used at higher doses, APAP is metabolized by several cytochrome p450 enzymes into the highly reactive metabolite *N*-acetyl-*p*-benzoquinoneimine (NAPQI) [31, 32], which is normally detoxified through conjugation with glutathione (GSH) both nonenzymatically and enzymatically in a reaction catalyzed by glutathione S-transferases (GSTs) [33]. In situations when sulfation and glucuronidation become saturated and cellular GSH production and conjugation systems are defective, excess NAPQI covalently binds to cellular macromolecules, resulting in oxidative stress and cytotoxicity [34]. Because Nrf2 can transcriptionally regulate genes that are responsible for the biotransformation and excretion of APAP, namely, UGT, glutamate cysteine ligase catalytic subunit (GCLC), glutamate-cysteine ligase modifier subunit (GCLM), GST, and NAD(P)H quinone oxidoreductase 1 (NQO-1) [6], Nrf2 activation is considered a prominent therapeutic target for APAP-induced hepatotoxicity (Figure 3). Two independent studies with Nrf2-knockout mice have shown that acetaminophen hepatotoxicity is exacerbated by Nrf2 deficiency. Large doses of APAP cause liver injury through oxidative stress, and Nrf2-knockout mice died sooner and at lower doses of APAP through the GSH synthesis pathway [35, 36]. Moreover, Reisman et al. reported that the elimination of APAP metabolites was decreased as a result of Mrp reduction in Nrf2-knockout mice. Furthermore, increased Mrp expression in Keap1-knockdown mice enhanced the efflux of APAP metabolites [37]. These results were confirmed by liver-specific Keap1-knockout mice which were significantly more resistant to toxic doses of acetaminophen than control animals which accompanied by the Nrf2 accumulation [10]. Goldring et al. reported that administration of APAP increased Nrf2 nuclear translocation in mouse liver as early as 60 minutes after treatment, with concomitantly increased expression of several downstream Nrf2 target genes [38]. Similar studies have confirmed the effect of APAP on hepatic induction of the Nrf2 target genes hemeoxygenase-1 (HO-1) and NQO-1 [39–41]. Elevation of NQO-1 is also observed in human liver tissues obtained during APAP overdose [42]. Moreover, NAPQI selectively modifies cysteine residues located within the intervening region of Keap1 [43]. It was recently reported that pharmacological stimulation of autophagy with rapamycin protects against APAP-induced hepatotoxicity [44]. Moreover, hepatic specific knockout of Atg5 mice, which is required specifically for autophagy, showed that persistent activation of Nrf2 and increased basal hepatic GSH levels and a faster recovery of GSH after APAP intoxication, which result in increased hepatocyte proliferation and protect against APAP-induced liver injury [45]. Furthermore, natural compounds with antioxidant activity such as isoliquiritigenin, sauchinone, oleanolic acid, and CDDO-Im protect against APAP-induced hepatotoxicity by activating Nrf2 [46–49]. 

### 3.2. Nrf2 Signaling in Chemical Hepatotoxin-Induced Hepatotoxicity

 Randle et al. reported the ability of chemical hepatotoxins such as bromobenzene, carbon tetrachloride (CCl_4_), and furosemide to induce hepatic Nrf2 nuclear translocation and Nrf2-regulated gene expression [50]. Repair of the liver injury after a single treatment with CCl_4_ was severely delayed in Nrf2-deficient mice [51]. 1-Bromopropane (1-BP), which is an alternative to ozone-depleting solvent, exhibited hepatotoxicity [52]. The Nrf2-knockout mice showed greater susceptibility to liver injury with reduced antioxidant response from 1-BP exposure compared to wild-type mice [53]. In contrast, Nrf2 activator such as curcumin attenuated dimethylnitrosamine-induced liver injury in rats [54]. For this reason, Nrf2 activation may be a therapeutic target for conditions that involve drug- or chemical hepatotoxin-induced hepatitis.

## 4. Role of Nrf2 in Alcoholic Steatohepatitis

 Alcoholic liver disease represents a broad spectrum of hepatic disorders, ranging from simple fatty liver (steatosis) to more severe forms of liver injury, including alcoholic steatohepatitis, cirrhosis, and HCC. Alcohol is a major contributor to liver disease-mediated deaths worldwide [55]. Alcohol is metabolized through several steps or pathways in the liver. First, alcohol dehydrogenase, cytochrome P450 2E1 (CYP2E1), and catalase metabolize alcohol to acetaldehyde, a highly toxic intermediate and well-known carcinogen. Then, acetaldehyde is further metabolized by aldehyde dehydrogenase to a less active byproduct called acetate. Increases in Nrf2 protein and mRNA levels were observed in liver tissues or hepatocytes from chronic alcohol-fed mice. HepG2 cells overexpressing CYP2E1 (E47 cells) showed increased Nrf2 mRNA and protein expression compared with control HepG2 (C34 cells). Nrf2 is activated in E47 cells as shown by an increase in nuclear translocation of Nrf2 and Nrf2-ARE binding activity and upregulation of Nrf2-regulated genes such as GCLC and HO-1 [56]. Lamlé et al. reported a critical role for Nrf2 in the protection against ethanol-induced liver injury. Nrf2-knockout mice given chronic ethanol administration showed significantly increased mortality associated with liver failure compared to wild-type mice. Reduced ability to detoxify acetaldehyde was detected in Nrf2-knockout mice, leading to accumulation of the toxic metabolite. Loss of Nrf2 caused a marked steatosis and inflammatory response mediated by Kupffer cells in ethanol-fed mice. Furthermore, chronic ethanol consumption led to a progressive depletion of total and mitochondrial reduced GSH, which was associated with more pronounced structural and functional changes to mitochondria of Nrf2-knockout mice [57]. Consistent with this, Nrf2 activation through Keap1 knockdown and hepatocyte-specific knockout blunted the increase in serum triglyceride and hepatic-free fatty acid in livers of ethanol-treated mice [58].

## 5. Role of Nrf2 in Nonalcoholic Steatohepatitis (NASH)

 Obesity and insulin resistance are highly associated with nonalcoholic fatty liver disease, which includes nonalcoholic fatty liver and NASH. Yates et al. carried out a global analysis of mouse hepatic gene expression and revealed that both genetic and pharmacologic activation of Nrf2 induce a larger cluster of genes associated with lipid metabolism [59]. Thus, Nrf2 activation seems to play an important role in energy metabolism, especially during the development and progression of fatty liver diseases. When mice are fed high-fat diets (HFDs), the mRNA levels of Nrf2 and its target genes are reduced in wild-type mice. However, another report showed that long-term feeding of HFD increased the mRNA level of Nrf2 [60]. Although Nrf2 expression after HFD feeding remains controversial, Nrf2 appears to be linked to metabolic liver diseases by diverse pathways. Severe liver injuries were observed in Nrf2-null mice compared to wild-type mice fed HFD [61]. Consistent with this, Nrf2-knockout mice exhibited a considerable increase in micro- and macrovesicular steatosis, and a massive increase in the number of neutrophil recruitments compared to those of wild-type mice when they are fed a methionine- and choline-deficient (MCD) diet. Livers of Nrf2-knockout mice fed MCD diet suffered more oxidative stress, iron accumulation, fibrosis and inflammation than wild-type mice [62–64]. Liver X receptor-*α* (LXR*α*), a member of the orphan nuclear receptor superfamily of ligand-activated transcription factors, regulates *de novo* fatty acid synthesis that stimulates hepatic steatosis [65, 66]. A recent report claimed that Nrf2 activation inhibits LXR*α* activity and LXR*α*-dependent liver steatosis. Increased hepatic steatosis parameters by treatment with LXR*α* synthetic ligand T0901317 were further enhanced by Nrf2 deficiency. Moreover, Nrf2 activator sulforaphane (SFN) inhibited T090-induced SREBP-1c and lipogenic genes in hepatocytes [67]. Kay et al. also showed that Nrf2 activation promoted deacetylation of farnesoid X receptor (FXR), inducing the FXR-target gene small heterodimer partner (SHP), which was responsible for LXR*α* repression. In agreement with that, the transcripts of LXR*α* and SREBP-1c were inversely correlated with those of Nrf2, FXR, and SHP in human samples of steatosis [67].

## 6. Role of Nrf2 in Cholestatic Liver Injury

**Figure 4:**
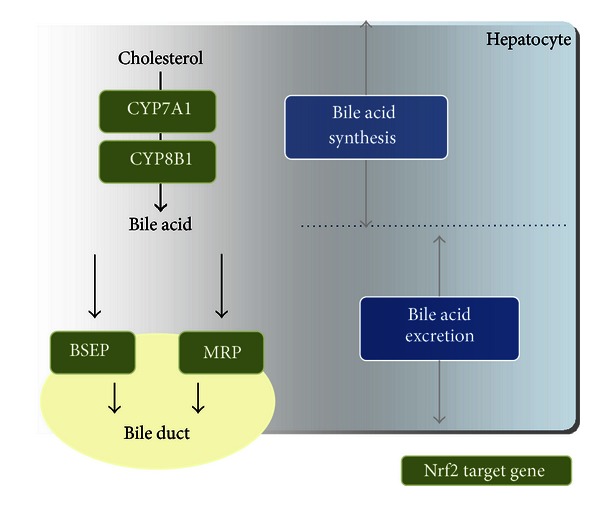
The role of Nrf2 target genes in bile acid homeostasis. Nrf2 regulates the synthesis and excretion of bile acid as a result of its target gene induction.

 Impaired hepatic bile flow can lead to excessive accumulation of toxic bile acids in liver cells, causing hepatic cholestasis and liver injury. The bile acid pumps, such as bile salt export pump and Mrp, are members of the ATP-binding cassette superfamily of transporters, and Nrf2 is a key regulator of induction of certain hepatobiliary transporters, as well as of hepatic detoxification and antioxidant mechanisms [68, 69]. Reduced rates of biliary bile acid, GSH excretion, and higher levels of intrahepatic bile acids were observed in Nrf2-knockout mice compared with wild-type mice after bile duct ligation (BDL). Moreover, the hepatic bile acid transporter gene expression was altered in Nrf2 deficiency. mRNA expression of efflux basolateral transporters such as Mrp3 and Mrp4 and bile acid synthetic enzymes CYP7a1 and CYP8b1 were reduced in Nrf2-knockout mice [70] (Figure 4). BDL is a useful animal model that leads to accumulation of bile acids in the liver and results in liver injury. However, Nrf2-knockout mice are not more susceptible to hepatic injury after BDL as shown by alanine aminotransferase (ALT) and histology data due to the compensatory response of bile acid transporters and nuclear receptors pregnane X receptor [70]. In contrast, BDL-induced liver injury is significantly attenuated in Keap1-knockdown mice compared with wild-type mice, through an enhancement of antioxidative stress systems, accompanied by Mrp efflux transport [71]. Lithocholic acid (LCA) is the most toxic secondary bile acid produced in the intestine, and elevated circulating levels of LCA induce cholestatic liver injury in rodents. Nrf2-knockout mice treated with LCA had significantly more severe multifocal liver necrosis compared with wild-type mice. This was accompanied by inflammation of bile ducts and necrosis of the ductal epithelium. Serum ALT and alkaline phosphatase levels were higher in Nrf2-knockout mice administered LCA than in wild-type mice [72]. Ursodeoxycholic acid (UDCA) improves clinical and biochemical indexes in a variety of cholestatic liver diseases [73]. UDCA significantly increased nuclear Nrf2 expression in livers of wild-type mice, and the treatment produced maximal hepatic induction of Mrp2, Mrp3, and Mrp4 in an Nrf2-dependent manner [74]. Moreover, UDCA treatment enhanced hepatic Nrf2 expression and phosphorylation and upregulated hepatic thioredoxin and thioredoxin reductase 1 protein expression in primary biliary cirrhosis patients [75]. These results indicate that Nrf2 activation is very useful for prevention or treatment of cholestatic liver injury.

## 7. Role of Nrf2 in Liver Fibrosis and Cirrhosis

A recent report showed that treatment with SFN inhibited the development and progression of early stage hepatic fibrosis induced by BDL in mice, accompanied by reduced expression of profibrogenic genes or hepatic stellate cell activation marker such as type I collagen or *α*-smooth muscle actin, respectively. In addition, SFN treatment suppressed transforming growth factor-*β*-induced Smad signaling and plasminogen activator inhibitor-1 expression [76]. After long-term CCl_4_ treatment, liver damage was also strongly aggravated in the Nrf2-knockout mice. Nrf2 deficiency enhanced and prolonged inflammatory and profibrogenic responses [51]. Further study is needed to define the role of Nrf2 and its molecular mechanisms in liver fibrosis and cirrhosis. 

## 8. Role of Nrf2 in HCC

HCC, one of the most frequent tumor types worldwide, results in over 1 million deaths per year. It is the fifth most common cancer and the third leading cause of cancer death [77]. Interestingly, many well-documented chemopreventive drugs from natural products have beneficial effects on suppression of carcinogenesis and many other chronic diseases through the activation of Nrf2. Recent studies with Nrf2-deficient mice demonstrated the role of Nrf2 in protecting liver from xenobiotic-mediated hepatocarcinogenesis. During long-term treatment of 2-amino-3-methylimidazo[4,5-*f*]quinoline, a carcinogenic and mutagenic heterocyclic amine derivative, the multiplicity and incidence of liver tumors in male and female were significantly higher in Nrf2-knockout mice than in wild-type mice [78]. Pomegranate, an ancient fruit with antioxidant properties, reduced hepatocarcinogenesis in the diethylnitrosamine, a dietary carcinogen, exposed rat model via Nrf2 upregulation and its target gene (e.g., hepatic antioxidant genes and carcinogen detoxifying enzymes) induction [79]. Nrf2 is thus regarded as a potential molecular target for cancer chemoprevention. 

## 9. Role of Nrf2 in Liver Regeneration

 Liver regeneration is a very complicated process orchestrated through a series of signaling cascades induced by cytokines, growth factors, and hormones [80]. In contrast to Nrf1-knockout mice, Nrf2-knockout mice were fertile and did not have developmental deficits [9], suggesting that Nrf2 is not required for development. But, it was recently reported that liver regeneration is significantly impaired in Nrf2-knockout mice after partial hepatectomy. Oxidative stress and hepatocyte apoptosis were enhanced after partial hepatectomy in Nrf2-knockout mice compared with those in wild-type mice in accordance with Nrf2-target gene repression. Nrf2 deficiency resulted in oxidative stress-mediated insulin/insulin-like growth factor resistance through impaired mitogen-activated protein kinases and Akt, which increased hepatocyte death and delayed proliferation [81]. The Notch signaling is essential for embryogenesis of mice and affects differentiation, proliferation, and apoptosis. Notch1 is activated and plays a crucial role in cell proliferation during liver regeneration after partial hepatectomy. Mouse embryonic fibroblast (MEF) cells isolated from wild-type and Nrf2-disrupted mice showed that Notch1 and its downstream target gene expression were repressed in Nrf2-knockout MEF cells. Furthermore, a functional ARE site was found in the promoter of Notch1. Nrf2-knockout mice showed lower levels of *Hes1 *transcripts, which reflect Notch1 signaling, following partial hepatectomy in Nrf2-knockout mice. However, constitutive expression of the gene encoding Notch1 and Notch1 signaling in the hepatocytes of Nrf2-knockout mice following partial hepatectomy enhanced liver regeneration to a level comparable to that in wild-type mice [82]. These results indicate that Nrf2 activation is essential for liver regeneration via alleviation of oxidative stress and regulation of hepatocyte proliferation. 

## 10. Conclusion

 Oxidative stress is implicated in the pathogenesis of liver disease. During periods of oxidative stress, Nrf2 is activated to protect the liver through target gene expression. Consistent with this, Nrf2-knockout mice are more susceptible to stresses such as chemical hepatotoxins. Moreover, most Nrf2 inducers are used for chemoprevention to detoxify carcinogens. These results show the pivotal role of the Nrf2-ARE pathway in liver pathophysiology. Moreover, they suggest the potential of Nrf2 as a therapeutic target to prevent and treat liver diseases.
